# Fungal Pathogens Associated with Aerial Symptoms of Avocado (*Persea americana* Mill.) in Tenerife (Canary Islands, Spain) Focused on Species of the Family Botryosphaeriaceae

**DOI:** 10.3390/microorganisms11030585

**Published:** 2023-02-25

**Authors:** David Hernández, Omar García-Pérez, Santiago Perera, Mario A. González-Carracedo, Ana Rodríguez-Pérez, Felipe Siverio

**Affiliations:** 1 Unidad de Protección Vegetal, Instituto Canario de Investigaciones Agrarias, 38270 San Cristóbal de La Laguna, Spain; 2 Servicio Técnico de Agricultura y Desarrollo Rural del Cabildo Insular de Tenerife, 38007 Santa Cruz de Tenerife, Spain; 3 Instituto Universitario de Enfermedades Tropicales y Salud Pública de Canarias, 38200 San Cristóbal de La Laguna, Spain; 4 Departamento de Bioquímica, Microbiología, Biología Celular y Genética, Universidad de La Laguna, 38200 San Cristóbal de La Laguna, Spain; 5 Sección de Laboratorio de Sanidad Vegetal, Consejería de Agricultura, Ganadería, Pesca y Aguas del Gobierno de Canarias, 38270 San Cristóbal de La Laguna, Spain

**Keywords:** *Lasiodiplodia brasiliensis*, *Neofusicoccum australe*, *Neofusicoccum cryptoaustrale*, *Neofusicoccum stellenboschiana*, *Neofusicoccum luteum*, *Neofusicoccum parvum*, *Persea americana*, dieback, canker, stem-end rot

## Abstract

Fungi of the family Botryosphaeriaceae are considered responsible for various symptoms in avocado such as dieback, external necrosis of branches and inflorescences, cankers on branches and trunks, or stem-end rot of fruits. In recent years, these problems are becoming more frequent in avocado orchards in the Canary Islands (Spain). This work includes the characterization of fungal species involved in these diseases, which were isolated from avocado crops in Tenerife Island between 2018 and 2022. A total of 158 vegetal samples were collected, from which 297 fungal isolates were culture-isolated. Fifty-two of them were selected according to their morphological features as representative isolates of Botryosphaeriaceae, and their molecular characterization was carried out, sequencing the ITS1-2 region as well as the *β-tubulin* and the *elongation factor 1-alpha* genes. Five species of Botryosphaeriaceae were isolated, including *Neofusicoccum australe, N. cryptoaustrale*/*stellenboschiana*, *N. luteum*, *N. parvum*, and *Lasiodiplodia brasiliensis*. This is the first time that *L. brasiliensis* has been associated with avocado dieback and that *N. cryptoaustrale*/*stellenboschiana* has been cited in avocado causing symptoms of dieback and stem-end rot. However, it was not possible to assign our isolates unequivocally to *N. cryptoaustrale* or *N. stellenboschiana* even additionally using the rpb2 marker for their molecular characterization. Botryosphaeriaceae family seem to be involved in avocado dieback, in the premature fall of fruits during their development in the field and in post-harvest damage in Tenerife, but further studies are needed to clarify the fungal pathogens associated with symptoms in relation to phenological plant growth stages or less frequently observed.

## 1. Introduction

Avocado (*Persea americana* Mill., 1768) is one of the most important crops in the Canary Islands, with the production area increasing at 5% per annum over the past decade. Plantations currently occupy about 2300 ha (more than 10% of its dedicated cultivation area in Spain) [1,2]. The avocado crops on the Canary Islands are affected by various diseases, with those caused by fungi of the Botryosphaeriaceae family more frequent over the past years but depending on the environmental conditions. In other avocado-producing regions, species of Botryosphaeriaceae have been associated with cankers and dieback on young and adult avocado plants, as well as with fruit rot (mainly stem-end rot) in post-harvest conditions (Table 1).

Symptoms of dieback are characterized by necrosis and death of twigs and branch-es in the tree canopy, with symptomatic plants exhibiting dry branches, external necrosis in twigs, and wilting leaves and inflorescences [3,4,5,6,7]. Cankers associated with Botryosphaeriaceae species (formerly *Dothiorella canker*) can occur on branches and trunks, where infections are initiated by spores entering through fresh wounds resulting from pruning, mechanical injury, sunburn or frost damage [8]. Cankers on avocado may exudate reddish-brown sap that turns to a whitish-yellowish powder when dry [8,9,10,11]. The bark appears darkened and friable and can be easily removed in old cankers. Underneath the bark, cankers are reddish-brown in colour and variable in shape, sometimes penetrating into the heartwood and affecting the xylem [9,10]; this may lead to dieback and collapse of branches or the entire tree [5,8,10]. In addition, fruit rot caused by Botryosphaeriaceae species is a common problem in avocado-producing regions (Table 1), representing an important threat to avocado production throughout the word. Symptoms of fruit rot are mostly developed after harvest when fruits begin to ripen. Then dark-coloured spots appear mainly in the insertion area of the peduncle (stem-end rot). As fruit ages, lesions gradually increase in size and become sunken, while decay spreads inside the fruit affecting the flesh that turns brown and watery [6,11,12,13,14]. Botryosphaeriaceae species associated with stem-end rot are present in cankers and living and dead branches and twigs collected from avocado trees [6,7,14], indicating that stem-end infections can be originated from endophytic fungal colonization. However, stem-end rot appears to be mostly originated from infections of peduncle that occur during fruit harvesting [7]. 

The Botryosphaeriaceae family (order *Botryosphaeriales*, *Ascomycota fungi*) includes numerous species. Some of them are morphologically similar, having only been differentiated and described in recent years by combining morphological characterization with phylogenetic analysis [15,16,17]. The species most frequently associated with avocado damage belong to the genera *Botryosphaeria*, *Diplodia*, *Dothiorella*, *Lasiodiplodia* and, especially, *Neofusicoccum* (Table 1). Fungal species of the Botryosphaeriaceae family are pathogenic to a wide range of woody hosts. They can also be found in nature as endophytes, behaving as latent or opportunistic pathogens that develop their pathogenicity when their hosts are subjected to stress conditions [18]. However, species of *Colletotrichum*, *Alternaria* and other genera, have also been described as causing similar symptoms, which sometimes could be erroneously attributed to fungal species of the Botryosphaeriaceae family [19].

In this work we show results of the studies carried out between 2018 and 2022 in Tenerife (Canary Islands, Spain), in an effort to obtain information about the symptoms attributed to Botryosphaeriaceae species in avocado orchards and post-harvest fruits. Morphological and molecular methods were used to characterize fungal isolates obtained from symptomatic samples, to determine the fungal agents associated with each symptomatology. Furthermore, pathogenicity of Botryosphaeriacerae species was tested on avocado seedlings.

## 2. Materials and Methods

### 2.1. Sampling and Fungal Isolation 

Between 2018 and 2022, 40 avocado orchards located in Tenerife (Canary Island, Spain), were surveyed for the presence of symptoms in the aerial parts of the trees. Orchards were selected based on information provided by Agricultural Extension Services (Council of Tenerife), or by technical advisors from local agricultural cooperatives who noticed symptoms of dieback of inflorescences, shoots and branches, branch or trunk cankers, or fruit rots. A total of 123 samples of necrotic panicles, shoots and branches, wood samples cut from cankers, or rotten avocado fruits were collected in the surveyed orchards. In addition, necrotic branches (11 samples), which were not located on the distribution maps, were directly provided by several avocado growers. Moreover, samples of postharvest fruits were obtained from two avocado packaging facilities (15 fruits), and from four retail stores (9 fruits). Samples were kept refrigerated at 5 °C until they were processed.

Vegetal fragments were taken with sterile scalpels from the transition zone between healthy and diseased tissue, disinfected with 0.5% sodium hypochlorite for 10 min, placed in 70% ethanol for 30 s, rinsed with sterile distilled water, and left to dry on sterile filter paper in a laminar flow cabinet. Afterwards, small pieces of about 5 mm^3^ were cut from the disease progression zones with a sterile scalpel, and fragments were plated in potato dextrose agar (PDA; Becton Dickinson, Sparks, MD, USA) plates, supplemented with 500–1000 mg/L Streptomycin (Sigma-Aldrich, St. Louis, MO, USA). Plates were incubated in the dark at 25 °C and examined for fungal growth over a 7-day period. Fungal colonies were isolated by transferring hyphal tips from the edge of the colonies to fresh PDA-Streptomycin (PDAS) plates (one isolate per plate). Isolates were preserved at −20 °C and −80 °C as mycelial plugs (5 mm diameter) in cryotubes containing 1 mL of 30% glycerol, as part of the fungal culture collection maintained at the Instituto Canario de Investigaciones Agrarias (ICIA, Canary Islands, Spain). 

### 2.2. Morphological and Molecular Identification of Fungal Isolates

Isolates were grown on PDA plates and examined for colony colour and growth pattern, mycelial morphology, as well as for production and morphology of reproductive structures. Isolates were grouped according to their morphological characteristics, in order to obtain a first tentative identification at genus level. Based on this preliminary characterization, representative isolates of Botryosphaeriaceae fungi, as well as other fungal species, were selected for molecular identification. Fungal isolates were grown on PDA plates for 7 days at 25 °C, and about 20 mm^2^ of mycelia was scraped off with a sterile scalpel. Recovered mycelia was grounded in an Eppendorf tube with a micropestle, and DNA was extracted with the EZNA Tissue DNA Purification Kit (Omega BIO-TEK, Norcross, GA, USA) or using a modification of the rapid HotSHOT extraction method of Truett et al. [34] as described in Collado-Romero et al. [35]. Genomic DNA extracts were stored at –20 °C until used.

Molecular identification of Botryosphaeriaceae species was carried out by PCR amplification and sequencing of the ribosomal DNA ITS1-5.8S-ITS2 region (ITS1-2), a region from the *translation elongation factor 1-alpha (tef1*) gene, and part of the *beta-tubulin* (*tub*2) gene, using primers pairs ITS-1/ITS-4 (5′-CTTGGTCATTTAGAGGAAGTAA-3′/5′-TCCTCCGCTTATTGATATGC-3′) [36], EF1-688F/EF1-986R (5′-CGGTCACTTGATCTACAAGTGC-3′/5′-TACTTGAAGGAACCCTTACC-3’) [37], and Bt2a/Bt2b (5′-GGTAACCAAATCGGTGCTGCTTTC-3′/5′-ACCCTCAGTGTAGTGACCCTTGGC-3′) [38], respectively. Additionally, and in order to contribute to the identification of the isolates obtained close to the species *N. cryptoaustrale* or *N. stellenboschiana*, PCR amplification and sequencing of their DNA extracts were carried out with the primers RPB2bot6F/RPB2bot7R (5′ GGTAGCGACGTCACTCCC-3′/5′-GGATGGATCTCGCAATGCG-3′) corresponding to the *rpb2* region [39]. Molecular identification of non-Botryosphaeriaceae fungal isolates was based only on the ITS1-2 region. 

PCR conditions for all amplifications consisted of an initial denaturation step (95 °C, 3 min), followed by 35 cycles of denaturation (95 °C, 30 s), annealing (53 °C, 30 s), and extension (72 °C, 1 min), and one final extension step (72 °C, 10 min). Each 25 µL PCR mix included 2.5 µL of 10× NH_4_ Buffer), 1.5 µL of 50 mM MgCl_2_, 0.25 µL of 100 mM dNTPs, 1.0 µL of 10 µM of each primer, 0.2 µL of 5 U/µL Taq DNA polymerase (VWR Life Science, Radnor, PA, USA), and 2.0 µL of DNA extract (substituted by 2.0 µL of H_2_O in negative control reactions). PCRs were incubated in a Mastercycler Gradient Thermocycler (Eppendorf AG, Hamburg, German). Amplicons were analysed in 1% agarose gels, prepared in 1× TAE (40 mM Acetate, 2 mM EDTA, 40 mM Tris-HCl, pH 8.0), and containing 1× RealSafe Nucleic Acid Staining (Durviz, Valencia, Spain). Gels were photographed under UV light using a UV transilluminator 2000 (Bio-Rad, Hercules, CA, USA), equipped with a Gel Logic 100 Imaging System (Kodak, Rochester, NY, USA). ExoSAP-IT PCR Product Cleanup (Applied Biosystems, Waltham, MA, USA) was used for enzymatic cleanup of PCR products, which were sequenced in both directions, with the same primers used for PCR amplification, at the *Servicio de Genómica* of the Universidad de La Laguna (Canary Islands, Spain). 

DNA sequences were manually inspected using the MEGA 11 package [40], to confirm basecalling quality, and edited to trim the tails. Next, a unique high-quality contig sequence was generated for each marker, after the alignment of forward and reverse reads. The contig sequence for each marker was independently used as query for a nucleotide BLAST search [41], against the NCBI GenBank nucleotide collection database (nr/nt) [42]. Species-level assignment was carried out considering the ten most closely related NCBI results, ordered by preference according to the minimum BLAST E-value. For species-level assignment, a minimum of 98% coverage and 98.5% identity percentage with a reference sequence was established as threshold. When reference sequences associated with different species were found inside the species-level assignment range, priority was established in base to published works. For Botryosphaeriaceae isolates, only those isolates identified as the same species independently using the three markers were considered as correctly characterized at species-level. 

Representative isolates of the different Botryosphaeriaceae species on the basis of BLAST searches were subjected to further morphological characterization. Isolates were cultured in PDAS medium, and mycelial plugs (5 mm diameter) were cut after 7 days, seeded in salt-cellulose medium enriched with sugarcane bagasse (SC) [43], and incubated for up to 42 days at 25 °C in the dark. This process was performed in Petri dishes sealed for 14 days with Parafilm M, and also in unsealed plates. Some of these isolates were also cultured in water agar medium amended with autoclaved pine needles (WA, 20 g/L Bacto^TM^Agar; (Becton Dickinson, Sparks, MD, USA)). These plates were incubated at 22 °C, 70% relative humidity and 12-h photoperiod, for 28 days. Plates were checked for pycnidia after 3 days, and then every 7 days. The time that each isolate took to produce pycnidia and conidia was recorded, and photographs of the cultures were taken. Pycnidia production and conidia characteristics were studied under a light microscope (Nikon Eclipse 80i) according to Marques et al. [44], Crous et al. [45], Yang et al. [46] and Phillips et al. [16]. The pycnidia were extracted from the medium and prepared on slides with 60% lactic acid. If the presence of conidia was detected, appearance, length and width were recorded for a total of 50 conidia per isolate. 

Phylogenetic trees were constructed independently for *Neofusiccocum* and *Lasiodiplodia* genera. ITS1-2, *tef1* and *tub2* gene sequences of representative isolates obtained in this work (Table 2) were compared with reference sequences downloaded from the NCBI database, including ex-type strains (Tables S1 and S2). Sequence alignments were obtained for each marker separately, using the ClustalW algorithm implemented in the MEGA 11 software [40]. Each alignment was manually inspected to confirm gap positions and trimmed from both ends to obtain a rectangular matrix dataset, prior to the concatenation of the three alignments.

The concatenated alignment for *Neofusicoccum* species included 29 reference ingroups and sequences from 46 strains isolated in the present study. *Botryosphaeria dothidea* CBS 115476 strain was included as outgroup. The three loci resulted in 1137 aligned sites. Among them, 43 positions corresponded with gaps, which were excluded from subsequent analysis. In the final dataset (1094 sites), a total of 165 positions were variable, being 78 of them parsimony-informative, and 87 singleton variants. The best substitution model was estimated for each marker independently, using IQ-Tree v.2.1.2 tool [47], at the CIPRES server [48], considering the Bayesian Information Criterion (BIC) value. For the three markers, the best substitution model was GTR + F [49], with BIC values of 2471.26 (ITS1-2), 1905.60 (*tef1*), and 1814.28 (*tub2*). For *Lasiodiplodia* species, the alignment included 24 reference ingroups, one strain from the present study, and *Neodeightonia phoenicum* CBS 122528 as outgroup. The three markers resulted in 1030 aligned sites. However, 61 positions were excluded since they corresponded with gaps. Therefore, the final dataset contains 969 sites, being 107 variables, 23 parsimony-informative, and 84 singletons variants. The best substitution model, estimated as described above, was GTR + F with BIC values of 1496.25, 1407.46, and 1480.50, for ITS1-2, *tef1* and *tub2*, respectively.

Maximum Likelihood (ML) and Bayesian Inference (BY) approaches were conducted for phylogenetic analyses, using the concatenated alignments. The ML and BY analyses were performed on the CIPRES server, using RAxML-HPC2 v.8.2.12 [50] and MrBayes v.3.2.7 [51], respectively. For the ML phylogeny, parameters were maintained as default, but 1000 bootstrap replicates were included. For BY analyses, the posterior probabilities (PP) were calculated by four Markov Chain Monte Carlo (MCMC) runs. Each chain included 5 × 10^6^ generations, and data were sampled every 100 generations. The first 12,000 calculations were discarded as the burn-in phase for each chain. The ML and BY trees of both genera were plotted using FigTree v.1.4.4 software, and topology of ML and BY trees were manually compared for congruence checking. As both approaches generate almost identical topologies, the BY tree was selected and ML bootstrap values were also included over the branches.

### 2.3. Pathogenicity Tests 

Pathogenicity of representative isolates of the different Botryosphaeriaceae species that were isolated in this study was tested by inoculating 6- to 12-month-old avocado seedlings (cv. Topa-topa or West Indian) grown at room temperature in pots (16.5 cm diameter × 36.5 cm height) with a volume of approximately 5 L, which were watered on demand. Avocado seedlings were inoculated with mycelial plugs (0.5 cm in diameter) of each isolate, previously grown on PDA for 7 days at 25 °C in the dark. Control plants were inoculated with sterile PDA discs without mycelium. Several pathogenicity tests were conducted throughout this study. Briefly, for plant inoculation with *L. brasiliensis* (isolate B161), *N. luteum* (isolate B153) and *N. parvum* (isolate B156), a wound of 0.5 cm in diameter was made with a sterile cork borer on the stem of each plant at 5 cm from the ground (6–8 plants per isolate), in which it was introduced a mycelial plug. The inoculated wounds were sealed with Parafilm M, which was removed after 15 days. Immediately after inoculation, half of the plants were bagged for 10 days with transparent plastic to increase the relative humidity. In subsequent tests, pathogenicity of representative isolates of *N. australe* (B018) and *N. crytoaustrale*/*stellenboschiana* (B043, B050), as well as of additional, representative isolates of *N. luteum* (B004, B047) and *N. parvum* (B034, B045, B113), was tested by pruning the seedlings at the top (2–8 plants per isolate), and then a mycelial plug was placed onto the pruning-cut wound and it was sealed with Parafilm M, which was removed after 4 days. Plants inoculated in this way were not bagged. At the end of the tests, pathogenicity was evaluated by examining the plants for external symptoms (dieback necrosis) and by cutting the stems longitudinally to assess necrotic inner lesions developed from the inoculation point. Koch’s postulates were confirmed after the reisolation from pieces of stem cut from the lesion margins as described above for fungal isolation.

## 3. Results

### 3.1. Sampling and Fungal Isolation

**Figure 1:**
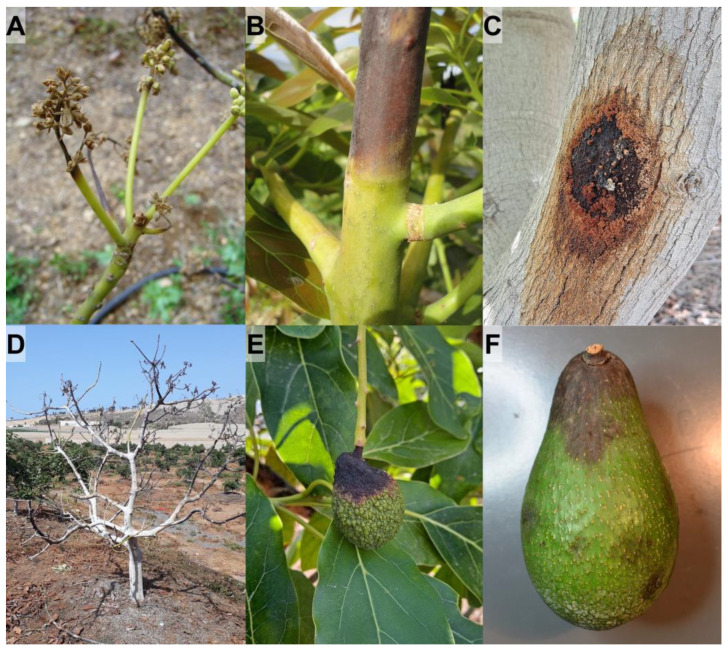
Symptoms usually observed in the aerial part of the avocado plants. (**A**), panicle blight; (**B**), branch dieback; (**C**), trunk canker; (**D**), collapse of entire young plant; (**E**), rots in developing fruits; (**F**), postharvest stem-end rot of fruit.

A total of 158 symptomatic avocado samples were studied in this work, from which 297 fungal strains were isolated. The analysed samples included 123 samples collected from 40 surveyed orchards; 11 samples of necrotic branches that were directly provided by growers; and 24 avocado fruits obtained from packaging facilities or stores. Symptoms usually observed in the aerial part of avocado plants included dieback, necrosis of twigs and inflorescences, or cankers on branches and trunks (Figure 1A–D). In addition, fruit rot was observed in postharvest, and also in developing fruits (Figure 1E,F). 

The most frequent symptom in the surveyed orchards was necrosis of branches (mainly diebacks), which was observed in 90.0% of them (Figure S1). Dieback represents a serious problem in new avocado plantations (Figure S1A,B), as necrosis spreads down both inside and outside of the small tender trunk from the apex, causing the necrosis above the graft, and subsequently the rootstock sprout out or die. Necrosis could continue to rot the branches downwards despite pruning them in healthy areas below the advance front of necrosis (Figure S1D–F), causing the collapse and death of the whole plant in the most severe cases (Figure S1C). Another symptom found in adult plantations was panicle dieback (25.0% of orchards), which results in necrosis of inflorescences (Figure S2A–D). Therefore, if necrosis affects a significant proportion of the panicles, avocado production could be greatly reduced. This necrosis also prevented the development of the shoots and could progress downwards, affecting the corresponding branch (Figure S2E–H) that become necrotic form the apex. Usually, the entire tree was not affected and therefore it remained productive in the healthy parts of the tree’s canopy. Cankers on branches and trunks were less frequent in affected plantations of the Canary Islands, as cankers were only observed in 7.5% of surveyed orchards. Sugary exudates could be seen in them, which once dry turns into a whitish-yellowish powder (Figure S3A–C), although avocado plants could produce the exudates for other reasons. In turn, the bark of the cankers could be cracked, dark in colour, or slightly sunken (Figure S3C). Beneath the canker, the inner bark and wood were brown, sometimes with reddish hues, instead of the normal pale colour. When the branch was cut transversely at the level of the canker, a wedge-shaped necrosis could be seen extending into the xylem (Figure S3D). In addition, fruit rot was observed in 12.5% of orchards. Symptoms of rot in developing fruits could appear either constricting the peduncle or directly affecting the fruit, which could detach or remains mummified hanging from the dry peduncle (Figure S3E–H). Regarding fruit rot in harvested avocados, they usually showed symptoms of stem-end rot, but also necrotic spots in other locations on the fruit (Figure S4). In postharvest, dark-coloured spots appeared mainly in the insertion area of the peduncle (stem-end rot), from where they gradually increase in size and can cover the entire surface of the fruit. Generally, the fungus also invaded the pulp (Figure S4), which began to discolour and gave a characteristic unpleasant odour when the fruit was opened for consumption.

### 3.2. Morphological and Molecular Identification of Fungal Isolates 

After a preliminary morphological characterization, 74 isolates showed morphological and growth features according to the Botryosphaeriaceae family, from which 52 were selected for their molecular identification based on the ITS1-2, *tef1* and *tub2* genetic markers. PCR amplification of ITS1-2, *tef1* and *tub2* markers gave products of 500–540 bp, 246–300 bp and 410 bp, respectively, and absence of contamination and non-specific PCR products were confirmed from agarose gels (not shown). Sequences obtained allowed the molecular identification of five different Botryosphaeriaceae species, based on independent BLAST searches with the three sequenced markers. One fungal isolate was identified as *Lasiodiplodia brasiliensis,* while four species of the *Neofusiccocum* genus were detected, including *N. australe* (2 isolates), *N. cryptoaustrale/stellenboschiana* (22 isolates), *N. luteum* (10 isolates), and *N. parvum* (12 isolates). The remaining five isolates were not identified to species level as they could not be recovered from storage. Twenty one out of the 22 isolates identified as *N. cryptoaustrale*/*stellenboschiana* with ITS1-2, *tef1* and *tub2* genomic regions showed for *rpb2* the same sequence (OQ401613, OQ401614 or Q401615), which matches all GenBank sequences from *N. stellenboschiana* and all-but-one *N. cryptoaustrale*. One isolate (B043) showed a sequence (OQ401617) equivalent to KX464014.1 which corresponds to that of the reference strain CBS 122813 of *N. cryptoaustrale*. These results were confirmed by repeating the analyses by means of a new DNA extraction using an alternative second procedure, PCR amplification and sequencing.

Colony growth, pycnidia and conidia of representative isolates of the different species of Botryosphaeriaceae identified in this work are shown in Figures S5–S9. All *Neofusiccocum* isolates generated pycnidia on SC medium with and without Parafilm M, except the isolate B022 of *N. parvum*, which only produced pycnidia on WA medium (Table 3). No conidia were obtained from *N. australe*, represented by isolate B018, but isolates of *N. cryptoaustrale*/*stellenboschiana* (B026), *N. luteum* (B003, B017, B024) *and N. parvum* (B020, B022) produced conidia on SC medium without Parafilm M and/or WA medium. In addition, the isolate B161, identified as *L. brasiliensis*, produced pycnidia and conidia on PDAS. Mature conidia of *L. brasiliensis* were dark-walled, one-septate, striate (Figure S9B), whereas conidia of *Neofusicoccum* species were hyaline and aseptate (Figures S6H, S7H and S8G). The conidia measurements are shown in Table 4, as well as reference measurements previously reported by other authors.

**Figure 2:**
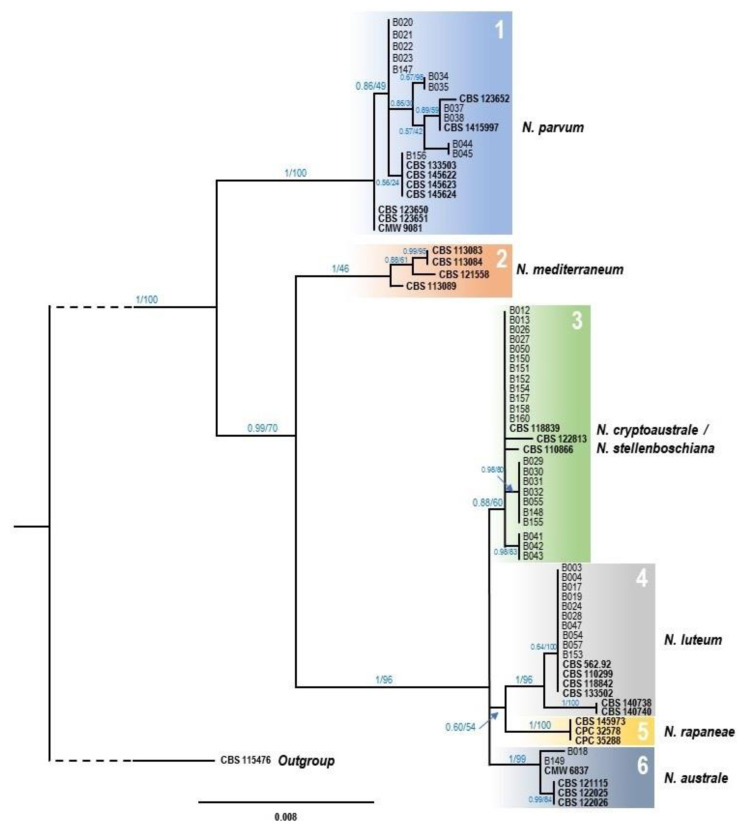
Phylogenetic tree of *Neofusicoccum* species resulting from a Bayesian analysis of the combined ITS1-2, *tef1* and *tub2* sequence alignment. Maximum likelihood bootstrap values and Bayesian posterior probabilities are shown at the nodes. Ex-type strains are indicated in bold font. The tree was rooted to *Botryosphaeria dothidea* (CBS 115476).

For the multiple alignment of *Neofusicocum* species, the ML and BY phylogenetic analysis generated identical topologies, showing six statistically well supported clades where the isolates from this study were placed in four of them. The BY tree is presented with PP/ML values at the corresponding branch (Figure 2). The first clade grouped twelve isolates from this study, along with the nine *N. parvum* reference sequences selected for the analysis, therefore confirming their molecular identification as *N. parvum* strains. The second clade, which includes the four reference sequences of *N. mediterraneum*, did not contain any isolate from the present work, suggesting that this species seems to be absent from the avocados sampled in Tenerife island. The third clade includes 22 of the new isolates along with *N. cryptoaustrale* CBS 122813 reference strain, but also with *N. stellenboschiana* CBS 110866 and CBS 118839. Interestingly, 12 of the isolates were placed in the main branch with the three sequences mentioned above, but the 10 remaining isolates were ubicated in two sub-clades, the first with seven isolates, and the second with three. The fourth clade corresponded with the *N. luteum* reference strains where ten isolates from this work were ubicated, all of them in the same group, along with reference strains CBS 562.92, CBS 110299, CBS 118842 and CBS 133502. Neither of the isolates of this study appeared in the fifth clade, corresponding this with *N. rapaneae*. Finally, the sixth and last clade grouped the two remaining isolates, along with *N. australe* reference strains.

**Figure 3:**
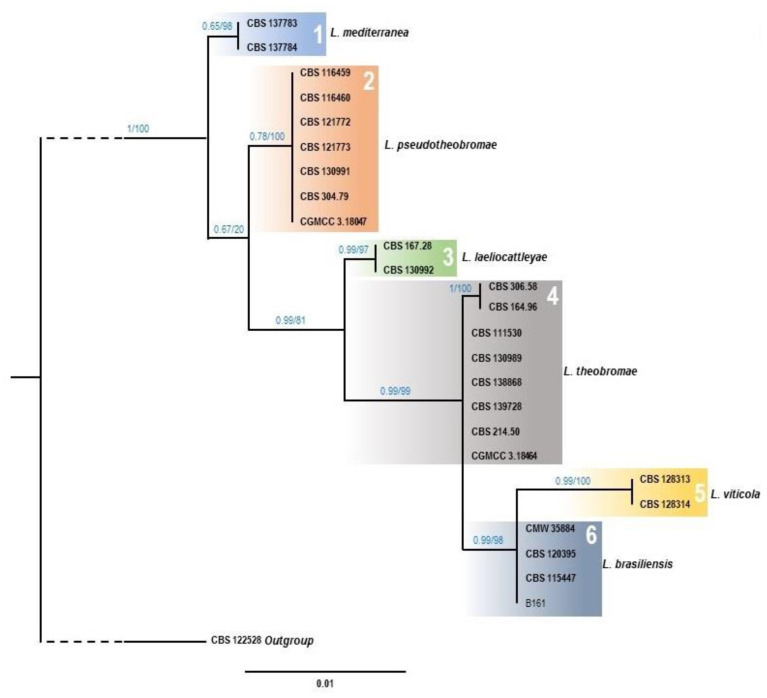
Phylogenetic tree of *Lasiodiplodia* species resulting from a Bayesian analysis of the combined ITS1-2, *tef1* and *tub2* sequence alignment. Maximum likelihood bootstrap values and Bayesian posterior probabilities are shown at the nodes. Ex-type strains are indicated in bold font. The tree was rooted to *Neodeightonia phoenicum* (CBS 122528).

In the case of *Lasiodiplodia* species ML and BY trees (Figure 3) showed exactly the same topology, showing six well supported clades corresponding to the species of *L. mediterranea*, *L. pseudotheobromae*, *L. laeliocattleyae*, *L. theobromae*, *L. viticola*, and *L. brasiliensis*. The unique isolate from the present work assigned to Lasiodiplodia genus (B161), was clearly assigned to the clade of *L. brasiliensis*.

### 3.3. Symptoms and Distribution of Botryosphaeriaceae Species Associated to Avocado in Tenerife

**Figure 4:**
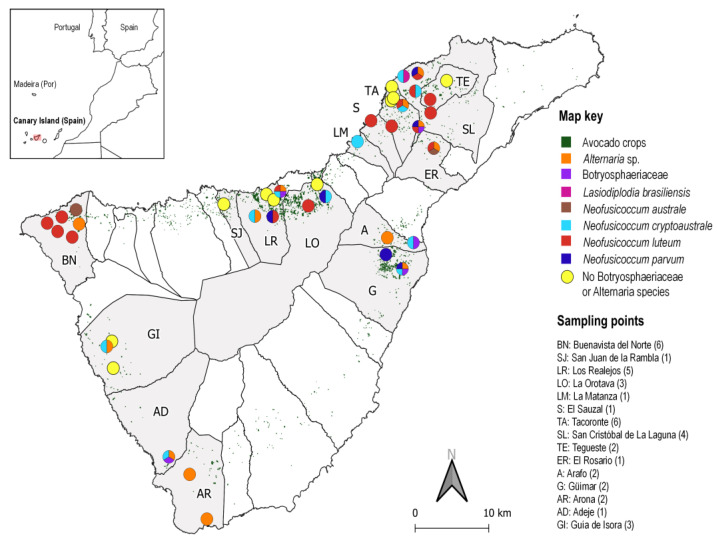
Map showing the sampling points and the species of fungi that were detected in Tenerife Island. Avocado plantations in the Canary Islands are highlighted in green. The map indicates the townships and the abbreviated names of places where samples were collected.

Some species of the Botryosphaeriaceae family were detected in 26 out of 40 orchards surveyed (Figure 4): *N. luteum*, 16 orchards; *N. cryptoaustrale/stellenboschiana*, 11 orchards; *N. parvum*, 6 orchards; *N. australe*, 2 orchards; *L. brasiliensis*, 1 orchard; and 5 orchards with unidentified species of Botryosphaeriaceae. There were also isolated species of *Alternaria* from 13 orchards (in four of them as the sole fungus, in the other nine along with Botryosphaeriaceae fungi). Other fungal species such as *Aureobasidium* sp., *Cladosporium* sp., *Colletotrichum* sp., *Nigrospora* sp. and *Pestalotiopsis* sp. were also isolated in 10 orchards.

Botryosphaeriaceae fungal species were isolated from samples of necrotic branches (mainly diebacks) and fruits collected in the surveyed orchards, with 47.6% and 30.3% of positive results, respectively. However, no Botryosphaeriaceae fungi were isolated from cankers and the isolates obtained from samples showing panicle blight were not species of Botryosphaeriaceae. In addition, *N. luteum* and *N. australe* were isolated from samples of necrotic branches that could not be geographically located, with the first species in two samples and the second in one. In samples of postharvest fruits collected in 6 sampling points (2 avocado packaging facilities and 4 retail stores), *N. luteum* was detected in 3 of them, *N. cryptoaustrale/stellenboschiana* in 2, and *N. parvum* and *N. australe* in 1 sampling point each one. 

### 3.4. Pathogenicity Tests

**Figure 5:**
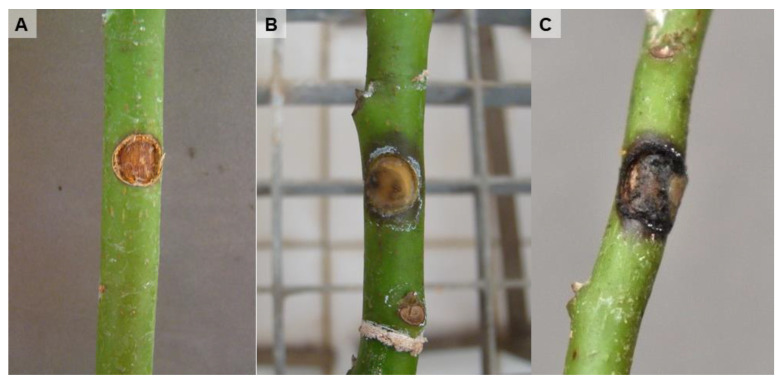
External symptoms in Topa Topa avocado seedlings 3 months after inoculation. Plants were inoculated by inserting a mycelial plug in a wound made in the stem at 5 cm from the ground. (**A**), negative control (plants inoculated with a fungus-free agar fragment); (**B**,**C**), damage caused by isolate B161 of *Lasiodiplodia brasiliensis*.

**Figure 6:**
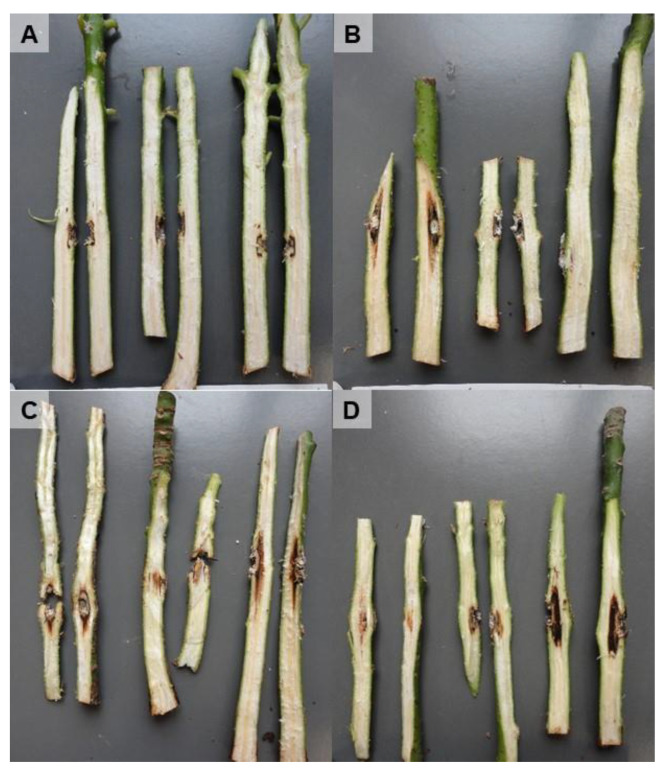
Internal symptoms in Topa Topa avocado seedlings 6 months after inoculation. Plants were inoculated by inserting a mycelial plug in a wound made in the stem at 5 cm from the ground. All plants shown in the figure were bagged for 10 d after inoculation. (**A**), negative control (plants inoculated with a fungus-free agar fragment); plants inoculated with isolates: (**B**), *Neofusicoccum luteum* B153; (**C**), *N. parvum* B156; and (**D**), *Lasiodiplodia brasiliensis* B161.

**Figure 7:**
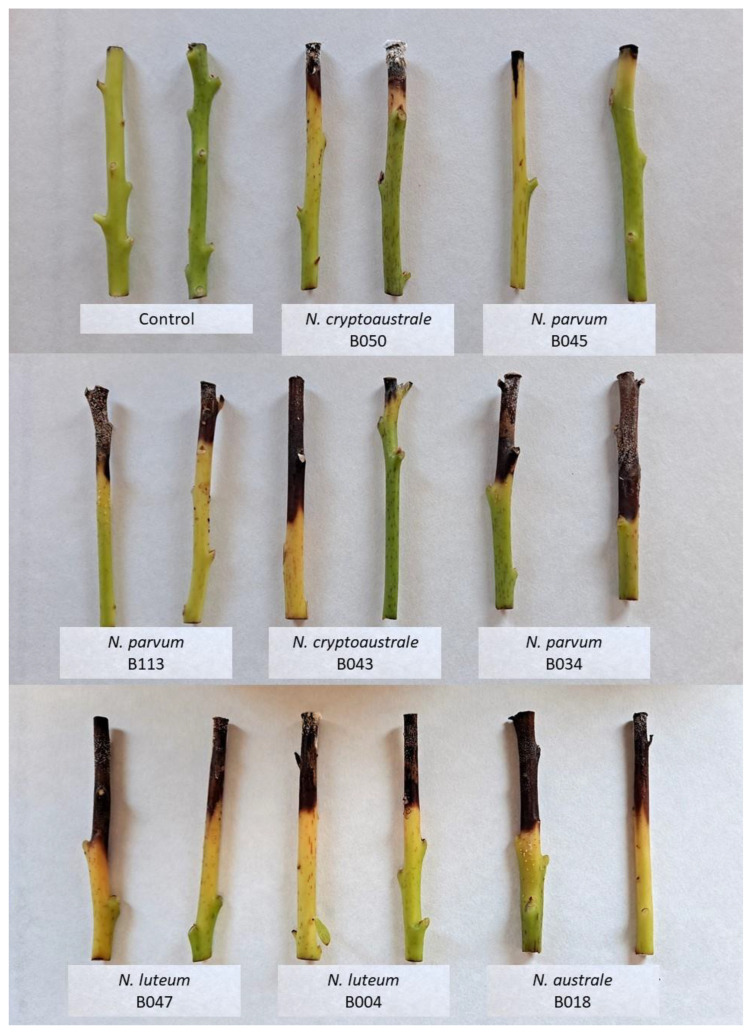
External symptoms in West Indian avocado seedlings 21 days after inoculation. Plants were pruned at the top, and they were inoculated by placing a mycelial plug onto the pruning-cut wound. Isolates B043, B050: *N. cryptoaustrale*/*stellenboschiana*. Negative control consists of plants inoculated with a fungus-free agar fragment.

Plants inoculated by inserting a mycelial plug in a wound made in the stem at 5 cm from the ground showed symptoms after 3–6 months being inoculated with *L. brasiliensis*, *N. luteum* and *N. parvum*. Figure 5 shows external stem necrosis produced by *L. brasiliensis*, with exudates of whitish sugars characteristic of avocado at the inoculation point or in the parts where Parafilm M was applied to fix the inoculum to the stem. All isolates caused internal necrosis that progressed from the inoculation point inside the plants, although irregularly and with variations in its length depending on the inoculated plant (Figure 6). No substantial differences were observed in the internal symptoms between bagged and non-bagged plants. When *N. australe*, *N. cryptoaustrale*/*stellenboschiana*, *N. luteum* and *N. parvum* were inoculated by pruning the plants at the top and placing the fungal inoculum onto the pruning-cut wound, external symptoms were clearly visible at 21 days after inoculation. Inoculated plants showed stem discolouration and necrosis that progressed along the outside of the plant stem downwards from the point of inoculation, along with the typical whitish-dry exudate (Figure 7, Table 5), showing the same symptoms of dieback that can be observed in the field. All the inoculated fungi in the different pathogenicity tests were re-isolated from the plant lesions, thus complying with Koch’s postulates.

## 4. Discussion

This work studied the occurrence of damages to the aerial part of avocado plants on the island of Tenerife and the diversity of the associated fungi. The symptom most frequently found was the necrosis in branches (dieback-type symptoms), which produces losses on plantlets in recent plantations and young trees, but also in adult trees. Panicle blight and fruit drop during its early development stages also caused significant losses in production. Cankers on trunks and branches appeared less frequently on field, although, very occasionally, some orchards may have many affected trees. We determined the presence and diversity of fungi associated with these symptoms using sequence analysis of ITS1-2, with particular interest in species of the Botryosphaeriaceae family, for which additional markers, *tef1* and *tub2*, were also used. 

Five species of Botryosphaeriaceae were isolated from symptomatic avocados: *Neofusiccocum australe*, *N. cryptoaustrale/stellenboschiana*, *N. luteum*, *N. parvum* and *Lasiodiplodia brasiliensis*, and it is important to highlight that it was frequent to find more than one species of this family in the same orchard. *Neofusicoccum australe*, *N. cryptoaustrale*/*stellenboschiana*, *N. luteum* and *N. parvum* have been reported in different countries as causal agents of diseases in avocado plants [5,6,7,10,33,52]. The high frequencies of *N. luteum* or *N. cryptoaustrale*/*stellenboschiana* found in this study does not correspond to what has been described in avocado-growing areas in the south of the Spanish mainland where *N. parvum* predominates [5]. No relationship was found in our study between the identified fungal species and the geographic location of avocado orchards. In other countries, the various types of symptoms seem to be caused in a general way by different species of the Botryosphaeriaceae family, along with other species of fungi, with the prevalence of one or the other according to countries or regions. Some publications even mention that the incidence and distribution of species varies between areas within the same region [5,10,53]. 

Moreover, fungi belonging to the Botryosphaeriaceae family have been isolated in the Canary Islands in other crops such as vineyards, almond trees, or mangoes [54,55], and in ornamental plants such as Indian laurels in which they cause significant damage in urban gardens [56]. The presence of Botryosphaeriaceae species infecting other hosts rather than avocado in the Canary Islands could play a role in the epidemiology of the diseases in avocado, as has been recently reported for other woody crops [57,58]. 

To the best of our knowledge, this study constitutes the first report of *N. cryptoaustrale*/*stellenboschiana* infecting avocado plants in Spain and the first time that *N. cryptoaustrale*/*stellenboschiana* has been cited in avocado causing symptoms of dieback and stem-end rot, although it had been previously cited causing branch cankers on avocado in Crete (Greece) [52]. However, it was not possible to assign our isolates unequivocally to one of these two species, as reference strains of *N. cryptoaustrale* and *N. stellenboschiana* are grouped in a unique clade. The additional analysis of the *rpb2* marker for *N. cryptoaustrale*/*stellenboschiana* showed that all analysed isolates except one presented a single sequence cited by various authors who have worked with this marker in the two species (the entire sequence fully matches for the two species). In addition, a sequence was obtained for isolate B043 that corresponded to the reference CBS:122813 registered in GenBank as KX464014.1. This sequence shows a difference of 24 snp with the others cited for *N. cryptoaustrale* and *N. stellenboschiana*. Therefore, in view of the results obtained it is necessary to clarify the taxonomic situation of both species and determine the origins of the differences, which is beyond the scope of this work. Moreover, results of conidial dimensions obtained for these isolates are similar to the references for both species, and they could not be used to morphologically discriminate them. Similar results were described by other authors who could not differentiate between *N. cryptoaustrale* and *N. stellenboschiana* using ITS1-2, *tef1* or t*ub2* markers [59,60]. 

*Neofusicoccum cryptoaustrale*/*stellenboschiana* seems to be quite widespread in avocado in Tenerife, considering the frequency with which it has been found in different locations (11 out of 40 orchards). *Neofusicoccum cryptoaustrale* has been isolated from branches and leaves of living *Eucalyptus* trees in South Africa [45], but also from other species of arboreal plants or other geographical locations such as *Pistacia* spp. [46,61], *Olea europea* [46] or different mangrove trees [62]. *Neofusicoccum stellenboschiana* was also recently described in South Africa isolated from *Vitis vinifera* [46], but also has been cited associated to *Arum italicum* leaf spot [46], *Olea europea* [46], *Persea americana* cankers [52], *Prunus* spp. [46] and *Quercus suber* [63]. Our pathogenicity tests carried out with isolates of this species show its ability to develop disease symptoms in avocado seedlings equivalent to those of *N. parvum* or *N. luteum*, which makes it a species to consider in the epidemiology of this disease in the Canary Islands. 

The unique isolate of the genus *Lasiodiplodia* obtained here was initially thought to belong to the most frequent species *L. theobromae*, already cited in avocado in several countries as well as in other plants in the Canary Islands [54,56]. However, its phylogenetic analysis indicates that it belongs to the species *L. brasiliensis*. This species was described in Brazil in 2014 associated with stem-end rot of papaya [64]. It has also been detected in that country associated to dieback in grapevine [65], postharvest fruit rot of custard apple [66] and cankers and dieback of apple trees [67]. Moreover, *L. brasiliensis* causes stem-end rot of mango in China [68]. It is closely related to *L. viticola,* but it differentiates on conidial sizes, which are longer and larger than those described for this last species [69]. Six nucleotides in the ITS1-2 region differentiate *L. brasiliensis* from *L. viticola,* with no difference in the nucleotide sequence of *tef1*. In our work, a single isolate of *L. brasiliensis* was obtained from an avocado plant with symptoms of dieback in an orchard in which *N. cryptoaustrale*/*stellenboschiana* was also detected. The results of the pathogenicity tests pointed out that *L. brasiliensis* was capable of reproducing symptoms of necrosis in inoculated avocado plants. This is the first time that this species has been reported in avocado and the first time that it has been reported in Spain. 

In this study, the number of samples of the main types of symptoms attributed to Botryosphaeriaceae in field conditions was highly unbalanced in favour of branch necrosis (mainly diebacks), with some representation of fruit rot and panicle blight and low representation of cankers on trunks or branches. According to the fungi isolated from these samples, the symptoms of branch necrosis (dieback-type) seem to be caused mainly by species of Botryosphaeriaceae, whereas fungi of other genus were prevalently isolated from panicle blight. No pathogens were consistently isolated from the few samples of cankers that were analysed. No fungal isolates from the Botryosphaeriaceae family were detected, nor were other species of harmful organisms to which cankers could be attributed such as *Phytophthora* species or bacteria (data not shown). Regarding the necrosis in the peduncles of unripe fruits that come off the trees without completing their development, it was possible to isolate species of Botryosphaeriaceae, *Alternaria* sp., but also other species. Although the loss of developing fruit is a natural process in the summer, we have to consider that stress, pests and diseases, such as those produced by Botryosphaeriaceae or other fungi, can cause excessive fruit drop. 

The same *Neofusicoccum* species found in avocado orchards (*N. australe*, *N. cryptoaustrale*/*stellenboschiana*, *N. luteum* and *N. parvum*) were isolated from post-harvest fruits showing symptoms of stem-end rot or dark spots in other locations on the fruit. In addition, *Colletotrichum* sp. was isolated only from one sample. It has been described that damages due to the Botryosphaeriaceae family are located in the area of insertion of the peduncle in the fruit (stem-end rot) [12,27,70], while those caused by *Colletotrichum* species are characterized by the appearance of darkening of the skin of the fruit (anthracnose) [12,27]. In this work, most of *Neofusicoccum* and *Colletotrichum* isolates were obtained from branches, suggesting that the fungi that infest the branches of avocado trees in Tenerife may also be the ones that cause damage to the fruit in post-harvest.

Regarding the pathogenicity tests, Koch’s postulates conducted with isolates of Botryosphaeriaceae obtained in this work confirmed their pathogenicity on avocado plants. Most of the inoculation procedures carried out with Botryosphaeriaceae fungi on avocado plants, but also in other woody plants, are mainly based on the use of a fungal mycelial plug, which is placed into a stem wound performed at a fixed distance from the ground and far from the top of the plant [3,6,9]. Using this procedure, inoculated plants show symptoms of necrosis several months after inoculation, and similar results were obtained in our study. However, when plants were inoculated by placing the inoculum onto pruning-cut wounds at the apex, inoculated plants showed symptoms of stem necrosis in less than a month. This procedure, based on wound inoculation at the apical tip region of the plants, has been described by some authors for pathogenicity tests of Botryosphaeriaceae fungi on avocado [4,11] and other woody plants [71], resulting in brown stem lesions 2–4 weeks after inoculation. Therefore, wound inoculation at the apex of the plants seems to reproduce disease symptoms in a short time, suggesting it could facilitate the study of different aspects of Botryosphaeriaceae disease on avocado such as varietal differences, variation in virulence between species and isolates, as well as to evaluate methods for its control.

In Tenerife, similarly to other avocado-growing regions, fungi of the Botryosphaeriaceae family seem to be involved in avocado dieback, in the premature fall of fruits during their development in the field, and in post-harvest damage. However, further research is needed in relation to panicle blight and avocado cankers in Tenerife. Panicle blight or inflorescence dieback occurs frequently and yields in some years are greatly reduced in the worst affected orchards, but little information is available on this symptom in avocado [19]. Fungi belonging to the family Botryosphaeriaceae have been described to cause cankers on avocado and other woody plants. However, no Botryosphaeriaceae fungi or other pathogens were consistently isolated from cankers in this work. The samples were taken from the cankers just in borders inside of it in adult trees without killing the tree, where other saprophytic organisms may have probably settled. In these old cankers, it is probably necessary to take the samples from more internal points at the zone of advancing necrosis. Furthermore, cankers are not frequent in avocado orchards in Tenerife and, for this reason, only a very low number of cankers could be analysed. Therefore, it is necessary to carry out a greater number of isolations, particularly from cankers, as well as from symptoms in relation to phenological growth stages to clarify their association with the fungal pathogens of avocado. 

## Figures and Tables

**Table 1:** Fungal species isolated from avocado plants or fruits showing symptoms usually attributed to fungi of the Botryosphaeriaceae family.

Country	Reference	Symptoms				Botryosphaeriaceae Species																	Non-Botryosphaeriaceae Species
		Plant		Fruit																			
		Branch or Trunk Canker	Dieback	Stem-End Rot	Black Spots	*Botryosphaeria dothidea*	*Diplodia mutila*	*Diplodia pseudoseriata*	*Diplodia seriata*	*Dothiorella aromatica*	*Dothiorella dominicana*	*Dothiorella iberica*	*Dothiorella* sp.	*Lasiodiplodia pseudotheobromae*	*Lasiodiplodia theobromae*	*Neofusiccocum australe*	*Neofusiccocum luteum*	*Neofusiccocum mangiferae*	*Neofusiccocum mediterraneum*	*Neofusiccocum nonquaesitum*	*Neofusiccocum parvum*	*Neofusiccocum* sp.	
Australia	[20]														X	X	X				X	X	
Australia	[12]			X	X	X				X	X												
Australia	[21]			X	X					X													Cg
Brasil	[22]			X	X	X															X		
Chile	[3]		X													X							
Chile	[23]			X												X							
Chile	[6]	X	X	X			X	X	X			X			X	X				X	X		
Chile	[24]			X	X												X						
China	[25]			X		X																	
Colombia	[26]	X	X												X								
Colombia	[27]			X	X								X		X								Cg, Phs, Pp, Ps
Ethiopia	[28]			X	X																		Af, An, Cg, Cls, Fs, Pes
Italy	[29]			X																	X		Cf, Cg,
Mexico	[30]				X																X		
New Zealand	[12]			X		X											X				X		Ca, Cg, Phs
New Zealand	[7]		X	X		X											X				X		Ca, Cg, Phs
Peru	[11]	X	X	X											X								As, Cos, Fs
South Africa	[13]			X	X					X													Cg, Cls, Np, Phs, Pms, Pp, Ps
Spain	[4]		X																		X		
Spain	[5]	X	X												X	X	X		X		X		Cg
Thailandia	[31]	X		X	X									X									
Taiwan	[32]				X													X					
Taiwan	[33]			X	X	X									X			X			X		
USA	[9]	X				X										X	X				X		
USA	[10]	X				X						X				X	X				X	X	Cos, Fs, Phs
USA	[8]	X										X				X	X				X		Phs
USA	[14]			X													X						Cg, Phs

Abbreviations: As, *Alternaria* spp.; Af, *Aspergillus flavus*; An, *Aspergillus niger*; Cls, *Cladosporium* spp.; Ca, *Colletotrichum acutatum*; Cf, *Colletotrichum fructicola*; Cg, *Colletotrichum gloeosporioides*; Cos, *Colletotrichum* spp.; Fs, *Fusarium* spp.; Np, *Nectria pseudotrichia*; Pes, *Pestalotiopsis* spp.; Phs, *Phomosis* spp.; Pms, *Phoma* spp.; Pp, *Pseudocercospora purpurea*; Ps, *Pestalotia* spp.

**Table 2:** GenBank accessions, representative isolates and groups of isolates with same sequences of *Neofusicoccum* and *Lasiodiplodia* species treated in the phylogenies.

			Genbank Accessions		
Species	Nº	Isolates ^1^	ITS	tef1	tub2
*N. australe*	2	**B018**, B149	OP788200	OQ236719	OQ181384
*N. cryptoaustrale/stellenboschiana*	12	**B012**, B013, B026, B027, B050, B150, B151, B152, B154, B157, B158, B160	OQ176234	OQ236715	OQ181395
	7	B029, B030, B031, B032, **B055**, B148, B155	OP788375	OQ236717	OQ181394
	3	**B041**, B042, B043	OP788376	OQ236716	OQ181393
*N. luteum*	9	**B003**, B004, B017, B019, B028, B047, B054, B057, B153	OP788373	OQ236713	OQ181386
	1	**B024**	OP788372	OQ236714	OQ181387
*N. parvum*	5	**B020**, B021, B022, B023, B147	OP788194	OQ236708	OQ181388
	2	**B034**, B035	OP788193	OQ236709	OQ181389
	2	**B037**, B038	OP788192	OQ236710	OQ181390
	2	**B044**, B045	OP788176	OQ236711	OQ181391
	1	**B156**	OQ176235	OQ236712	OQ181392
*L. brasiliensis*	1	**B161**	OP788199	OQ236718	OQ181385

^1^ Representative isolates submitted to GenBank are in bold.

**Table 3:** Production of pycnidia/conidia by *Neofusicoccum* isolates.

Species	Isolate	Apparition of Pycnidia/Conidia (Days)		
		SC-P	SC	WA
*N. australe*	B018	14/NP	21/NP	14/NP
*N. cryptoaustrale*/*stellenboschiana*	B012	14/NP	14/NP	ND
	B026	14/NP	14/NP	14/21
	B030	21/NP	14/NP	ND
*N. luteum*	B003	21/NP	21/42	14/21
	B017	14/NP	14/14	ND
	B024	14/NP	14/28	ND
*N. parvum*	B020	28/NP	28/42	14/NP
	B022	NP/NP	NP/NP	21/21

SC-P: salt-cellulose agar medium + sugarcane bagasse sealed first 14 days with Parafilm M; SC: salt-cellulose agar medium + sugarcane bagasse without Parafilm M; WA: water-agar medium + pine needles; ND, not determined; NP, not produced.

**Table 4:** Conidial dimensions of *Lasiodiplodia* and *Neofusicoccum* isolates from this study and comparison with previous reports.

Species	Isolates	Values Obtained in This Study			References	
		Length (L) (µm)	Width (W) (µm)	L/W Ratio	Length (L) (µm)	Width (W) (µm)
*L. brasiliensis*	B161	23.49 ± 1.42	14.95 ± 1.18	1.57	25.1–27.3 ^1^	13.3–14.79 ^1^
*N. cryptoaustrale*/*stellenboschiana*	B026	20.44 ± 1.41	6.02 ± 0.51	3.4	(18–) 20.5–21 (–26.5) ^2^ (17–) 19–21 (−22) ^3^	5–6 (–6.5) ^2^ (4.5–) 5.5–6 ^3^
*N. luteum*	B003	18.60 ± 2.72	5.93 ± 0.60	3.14	(15–) 16.5–22.5 (–24) ^4^	4.5–6 (–7.5) ^4^
	B017	21.63 ± 1.55	6.34 ± 0.69	3.41		
	B024	18.34 ± 1.56	5.50 ± 0.38	3.33		
*N. parvum*	B020	14.97 ± 1.05	5.70 ± 0.65	2.63	(12–) 13.5–21 (–24) ^4^	4–6 (–10) ^4^

The sizes of the conidia were taken from: ^1^ *L. brasiliensis* from Marques et al. [44], ^2^ *N. cryptoaustrale* from Crous et al. [45], ^3^ *N. stellenboschiana* from Yang et al. [46] and ^4^ *N. luteum* and *N. parvum* from Phillips et al. [16].

**Table 5:** External symptoms (length of necrotic lesion) caused by *Neofusicoccum* species in West Indian avocado seedlings 21 days after inoculation in pruning-cut wound.

Species	Isolate	Mean Lesion Length (cm)	Standard Deviation (cm)
Control	-	0.0	0.0
*N. australe*	B018	4.8	0.2
*N. cryptoaustrale*/*stellenboschiana*	B043 ^1^	3.7/2.5	3.5/1.3
	B050	2.4	0.3
*N. luteum*	B004	4.3	0.3
	B047	5.1	1.2
*N. parvum*	B034	5.6	1.2
	B045	1.4	0.7
	B113 ^1^	3.4/3.9	0.8/2.7

^1^ Pathogenicity tests with isolates B043 and B113 were repeated twice.

## Data Availability

Not applicable.

## Supplementary Materials

The following supporting information can be downloaded at: https://www.mdpi.com/article/10.3390/microorganisms11030585/s1, Figure S1: Symptoms in aerial parts of young and adult avocado plants; Figure S2: Panicle blight and dieback symptoms; Figure S3: Cankers in blanch and trunks, and rots in developing fruits; Figure S4: Symptoms in post-harvest avocado fruits; Figure S5. *Neofusiccocum australe*; Figure S6. *Neofusiccocum cryptoaustrale*/stellenboschiana; Figure S7. *Neofusiccocum luteum*; Figure S8. *Neofusiccocum parvum*; Figure S9. *Lasiodiplodia brasiliense*. Table S1. GenBank and culture collection accession numbers of *Neofusicoccum* species treated in the phylogenies. Table S2. GenBank and culture collection accession numbers of *Lasiodiplodia* species treated in the phylogenies.
