# Supplementary material for: Fungal Pathogens Associated with Aerial Symptoms of Avocado (Persea americana Mill.) in Tenerife (Canary Islands, Spain) Focused on Species of the Family Botryosphaeriaceae
Source: Microorganisms. 2023 Feb 25;11(3):585. doi: 10.3390/microorganisms11030585 (PMC10058760; doi:10.3390/microorganisms11030585)
Supplement: Supplementary file 1 [file microorganisms-11-00585-s001.zip › Table S1.pdf]

Table S1. GenBank and culture collection accession numbers of *Neofusicoccum* species treated in the phylogenies

Species name	Isolated/Culture collection number[1]	Host	Country	Collector(s)	GenBank accession numbers		
					ITS1-2	*tef1*	*tub2*
Botryosphaeria dothidea	CBS 115476	*Prunus* sp.	Switzerland	B. Slippers	AY236949	AY236898	AY236927
N. australe	CBS 121115	*Prunus persica*	South Africa	U. Damm	EF445355	EF445386	KX464948
	CBS 122025	*Eucalyptus* sp.	Spain	R. Alzugaray	KX464160	KX464672	KX464949
	CBS 122026	*Eucalyptus* sp.	Spain	R. Alzugaray	KX464161	KX464673	KX464950
	**CMW 6837**	*Acacia* sp.	Australia	M.J. Wingfield	AY339262	AY339270	AY339254
N. cryptoaustrale	**CBS 122813**	*Eucalyptus* sp.	South Africa	H.M. Maleme	FJ752742	FJ752713	FJ752756
N. luteum	**CBS 562.92**	*Actinidia deliciosa*	New Zealand	S.R. Pennycook	KX464170	KX464690	KX464968
	CBS 110299	*Vitis vinifera*	Portugal	A.J.L. Phillips	AY259091	KX464688	DQ458848
	CBS 118842	*Syzygium cordatum*	South Africa	D. Pavlic	DQ316088	MT592196	MT592688
	CBS 133502	*Persea americana*	USA	A. Eskalen	MT587483	MT592197	MT592689
	CBS 140738	*Avicennia marina*	South Africa	J.A. Osorio & J. Roux	NR_147360	MT592206	MT592698
	CBS 140740	*Bruguieria gymnorrhiza*	South Africa	J.A. Osorio	MT587493	MT592208	MT592700
N. mediterraneum	**CBS 113083**	*Pistacia vera*	USA	T.J. Michailides	KX464186	KX464712	KX464998
	CBS 113084	*Sequoia sempervirens*	USA	T.J. Michailides	KX464187	KX464713	KX464999
	**CBS 113089**	*Pistacia vera*	USA	T.J. Michailides	KX464199	KX464727	KX465014
	CBS 121558	*Olea europea*	Italy	F. Salvatore	GU799463	GU799462	GU799461
N. parvum	CBS 123650	*Syzygium cordatum*	South Africa	D. Pavlic	KX464182	KX464708	KX464994
	CBS 123651	*Syzygium cordatum*	South Africa	D. Pavlic	KX464183	KX464709	KX464995
	CBS 123652	*Syzygium cordatum*	South Africa	D. Pavlic	KX464184	KX464710	KX464996
	CBS 133503	*Persea americana*	USA	A. Eskalen	MT587504	MT592219	MT592711
	CBS 145622	*Ficus carica*	Italy	D. Aiello	MN611179	MN623346	MN623343
	CBS 145623	*Ficus carica*	Italy	D. Aiello	MN611180	MN623347	MN623344
	CBS 145624	*Ficus carica*	Italy	D. Aiello	MN611181	MN623348	MN623345
	CBS 145997	*Aloe* sp.	South Africa	P.W. Crous	MT587449	MT592159	MT592649

Table S1. GenBank and culture collection accession numbers of *Neofusicoccum* species treated in the phylogenies

Species name	Isolated/Culture collection number[1]	Host	Country	Collector(s)	GenBank accession numbers		
					**ITS1-2**	***tef1***	***tub2***
	**CMW 9081**	*Populus nigra*	New Zealand	G.J. Samuels	AY236943	AY236888	AY236917
N. rapaneae	**CBS 145973**	*Rapanea melanophloeos*	South Africa	M.J. Wingfield	MT587511	MT592226	MT592718
	CPC 32578	*Rapanea* sp.	South Africa	M.J. Wingfield	MT587512	MT592227	MT592719
	CPC 35288	*Rapanea* sp.	South Africa	M.J. Wingfield	MT587513	MT592228	MT592720
N. stellenboschiana	CBS 110866	*Vitis vinifera*	South Africa	F. Halleen	MT587518	MT592242	MT592734
	CBS 118839	*Syzygium cordatum*	South Africa	D. Pavlic	DQ316085	MT592243	MT592735

[1] Bold culture collection numbers means ex-type strains
