# Supplementary material for: Fungal Pathogens Associated with Aerial Symptoms of Avocado (Persea americana Mill.) in Tenerife (Canary Islands, Spain) Focused on Species of the Family Botryosphaeriaceae
Source: Microorganisms. 2023 Feb 25;11(3):585. doi: 10.3390/microorganisms11030585 (PMC10058760; doi:10.3390/microorganisms11030585)
Supplement: Supplementary file 1 [file microorganisms-11-00585-s001.zip › Table S2.pdf]

Table S2. GenBank and culture collection accession numbers of *Lasiodiplodia* species treated in the phylogenies.

Species name	Isolated/Culture collection number[1]	Host	Country	Collector(s)	GenBank accession numbers		
					**ITS1-2**	***tef1***	***tub2***
L. brasiliensis	CBS 115447	*Psychotria tutcheri*	Hong Kong, China	-	MT587422	MT592134	MT592614
	CBS 120395	*Theobroma cacao*	Cameroon	M. Mbenoun & N. Amoudou	MT587423	MT592135	MT592615
	CMW 35884	*Adansonia madagascariensis*	Madagascar	-	KU887094	KU886972	KU887466
L. laeliocattleyae	**CBS 130992**	*Mangifera indica*	Egypt	A. Ismail	JN814397	JN814424	KU887508
	**CBS 167.28**	*Laelio cattleya*	Italy	C. Sibilia	MT587425	MT592136	MT592618
L. mediterranea	**CBS 137783**	*Quercus ilex*	Italy	B.T. Linaldeddu	KJ638312	KJ638331	KU887521
	CBS 137784	*Vitis vinifera*	Italy	S. Serra	KJ638311	KJ638330	KU887522
L. pseudotheobromae	CBS 116459	*Gmelina arborea*	Costa Rica	J. Carranza-Velásquez	EF622077	EF622057	EU673111
	CBS 116460	*Acacia mangium*	Costa Rica	-	EF622078	EF622058	KU198428
	CBS 121772	*Acacia mellifera*	Namibia	F.J.J. van der Walt & J. Roux	EU101310	EU101355	MT592627
	CBS 121773	*Acacia mellifera*	Namibia	F.J.J. van der Walt & J. Roux	EU101311	EU101356	MT592628
	CBS 130991	*Mangifera indica*	Egypt	A. Ismail	MT587433	MT592145	MT592629
	CBS 304.79	*Rosa cv. Ilona*	Netherlands	P.D. Wageningen	EF622079	EF622061	MT592630
	CGMCC 3.18047	*Pteridium* sp.	China	W. He & Z. Dou	KX499876	KX499914	KX499989
L. theobromae	CBS 111530	*Leucospermum* sp.	USA	J.E. Taylor	EF622074	EF622054	KU887531
	CBS 130989	*Mangifera indica*	Egypt	A.M. Ismail	MT587437	MT592149	MT592634
	CBS 138868	*Ficus religiosa*	Iran	M. Mirabolfaty	MT587438	MT592150	MT592635
	CBS 139728	Human, knee	Netherlands	N. Desbois	MT587439	MT592151	MT592636
	**CBS 164.96**	Fruit along coral reef coas	Papua	A. Aptroot	AY640255	AY640258	KU887532
	CBS 214.50	*Cajanus cajan*	India	-	MT587440	MT592152	MT592637
	CBS 306.58	*Cocos nucifera*	-	-	EF622071	EF622051	MT592638
	**CGMCC 3.18464**	*Aquilaria crassna*	Laos	X. Sun	KY783471	KY848609	KY848552
L. viticola	**CBS 128313**	*Vitis vinifera*	USA	R.D.Cartwright & W.D. Gubler	HQ288227	HQ288269	HQ288306
	CBS 128314	*Vitis vinifera*	USA	K. Striegler & W.D. Gubler	HQ288228	HQ288270	HQ288307
Neodeightonia phoenicum	CBS 122528	*Phoenix* sp.	Spain	F. Garcia	EU673340	EU673309	EU673116

[1] Bold culture collection numbers means ex-type strains
