# Supplementary figures and images for: Fungal Pathogens Associated with Aerial Symptoms of Avocado (Persea americana Mill.) in Tenerife (Canary Islands, Spain) Focused on Species of the Family Botryosphaeriaceae
Source: Microorganisms. 2023 Feb 25;11(3):585. doi: 10.3390/microorganisms11030585 (PMC10058760; doi:10.3390/microorganisms11030585)

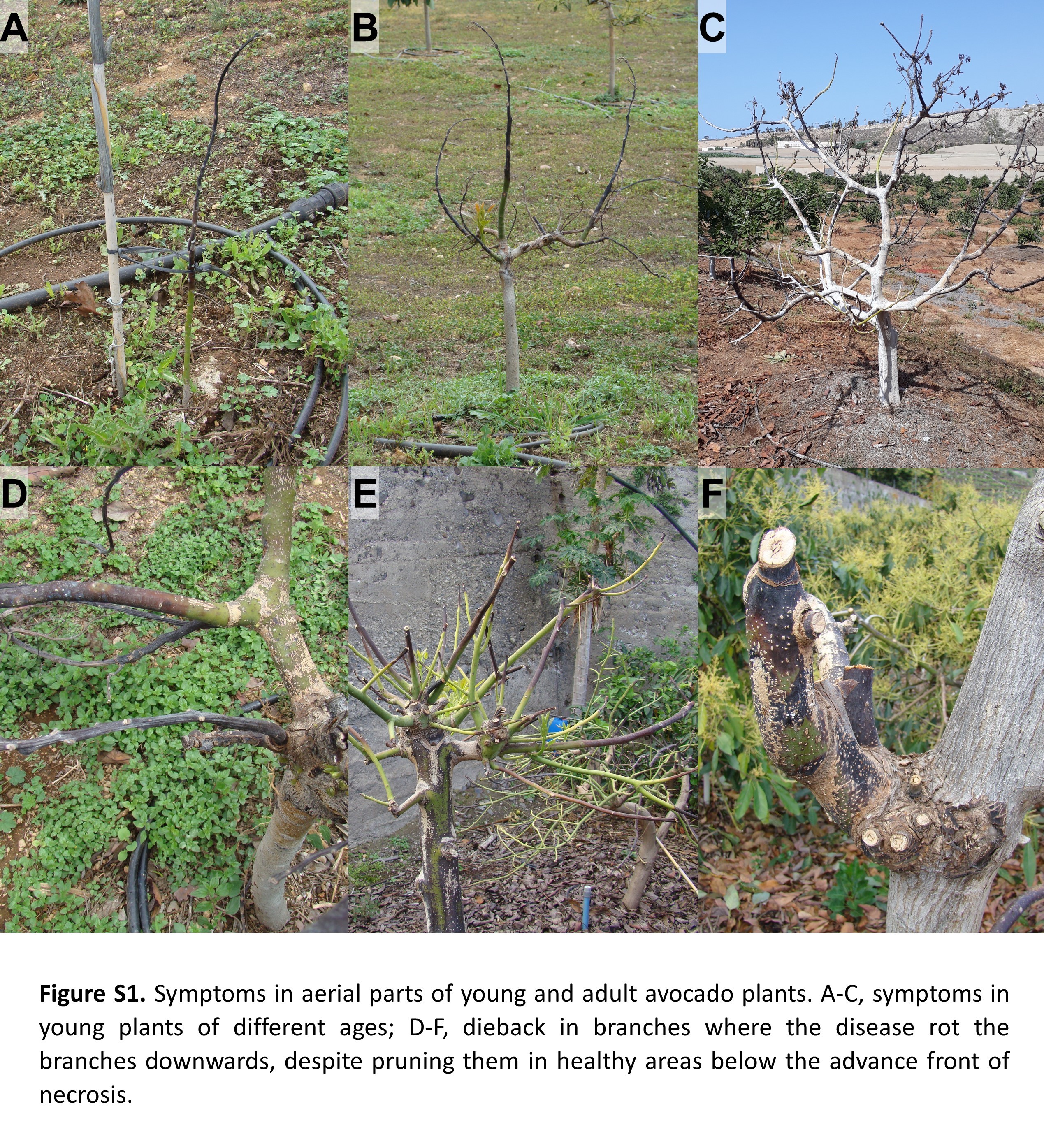

Supplement: Supplementary file 1 [file microorganisms-11-00585-s001.zip › Figure S1.jpg]

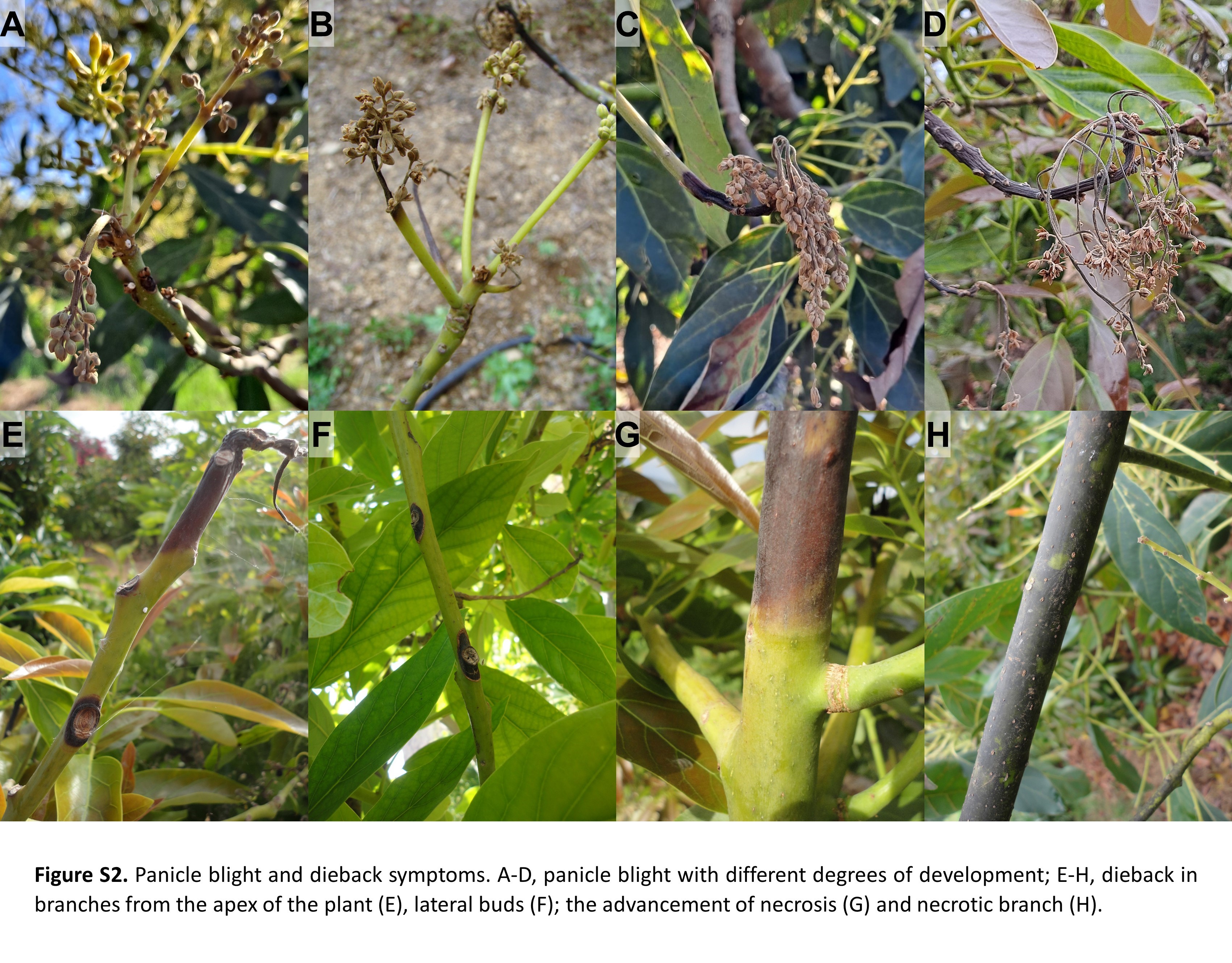

Supplement: Supplementary file 1 [file microorganisms-11-00585-s001.zip › Figure S2.jpg]

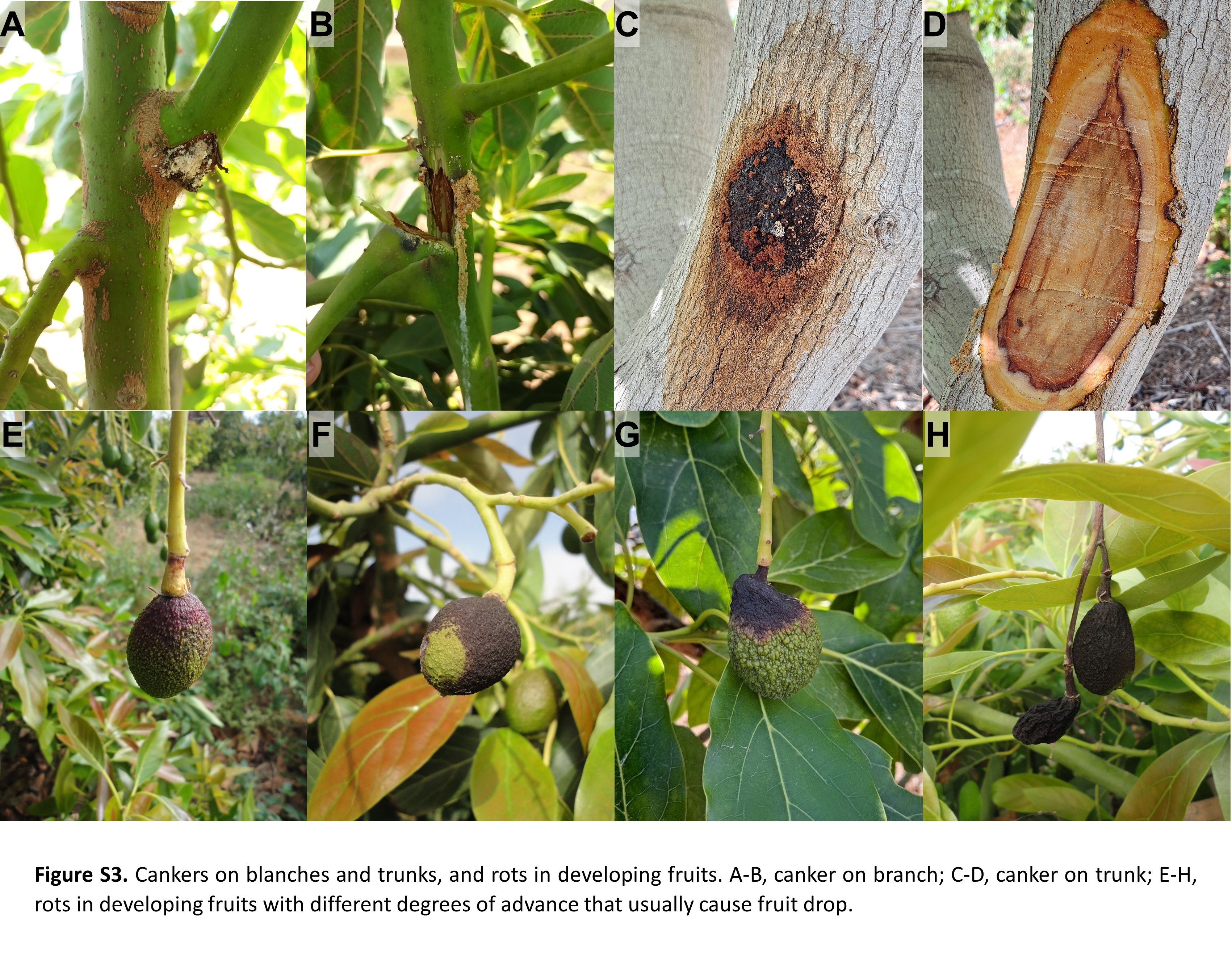

Supplement: Supplementary file 1 [file microorganisms-11-00585-s001.zip › Figure S3.jpg]

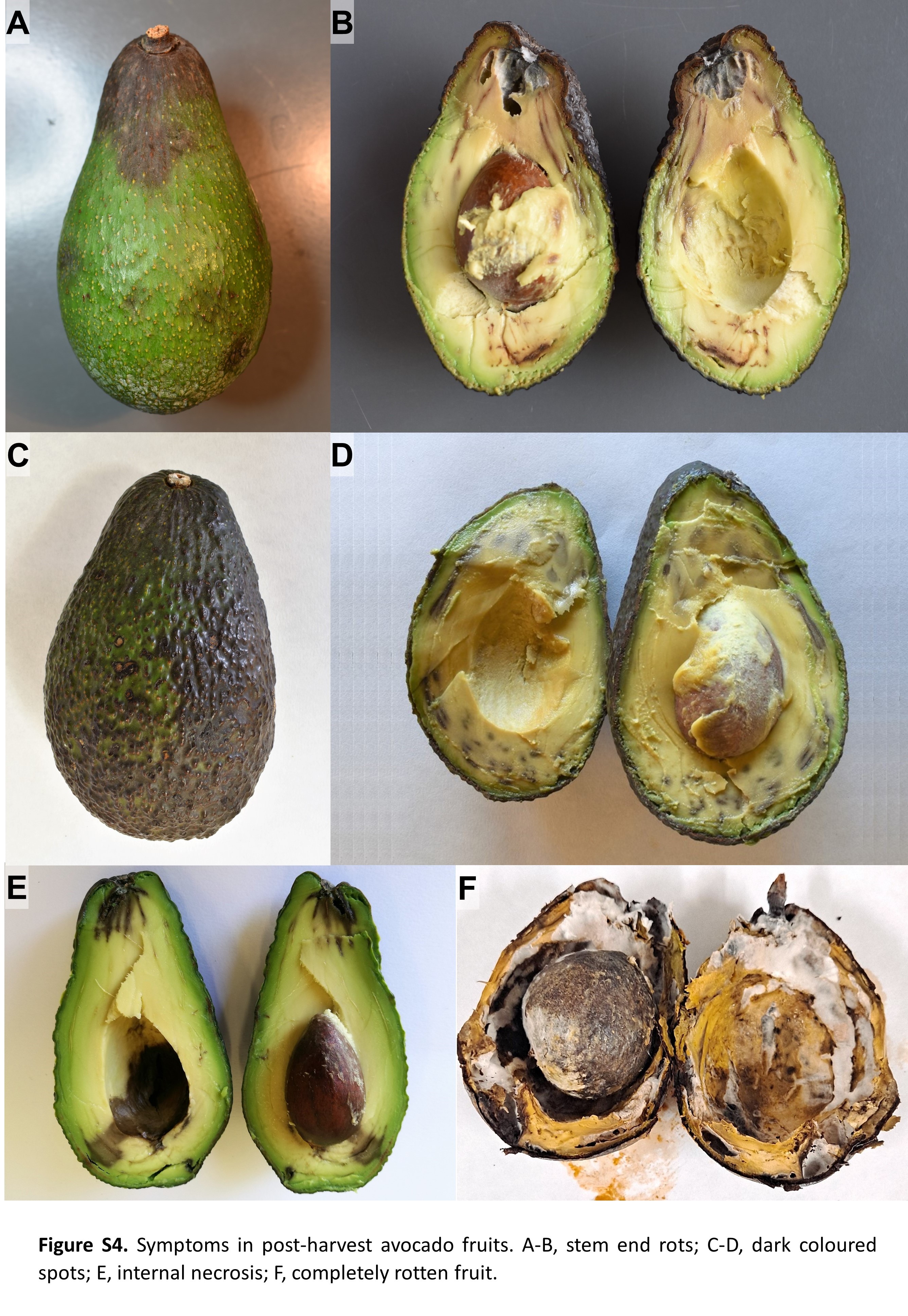

Supplement: Supplementary file 1 [file microorganisms-11-00585-s001.zip › Figure S4.jpg]

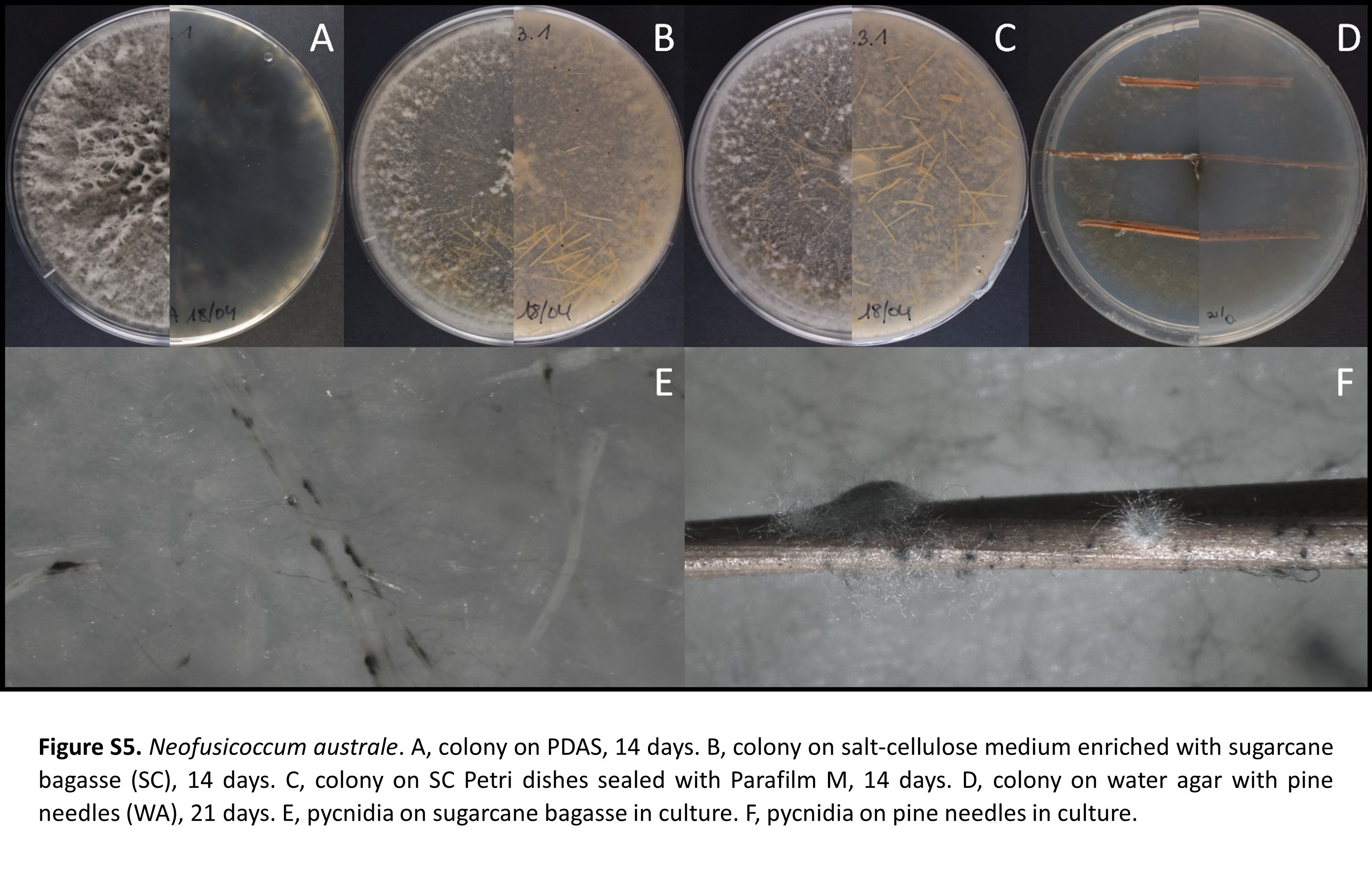

Supplement: Supplementary file 1 [file microorganisms-11-00585-s001.zip › Figure S5.jpg]

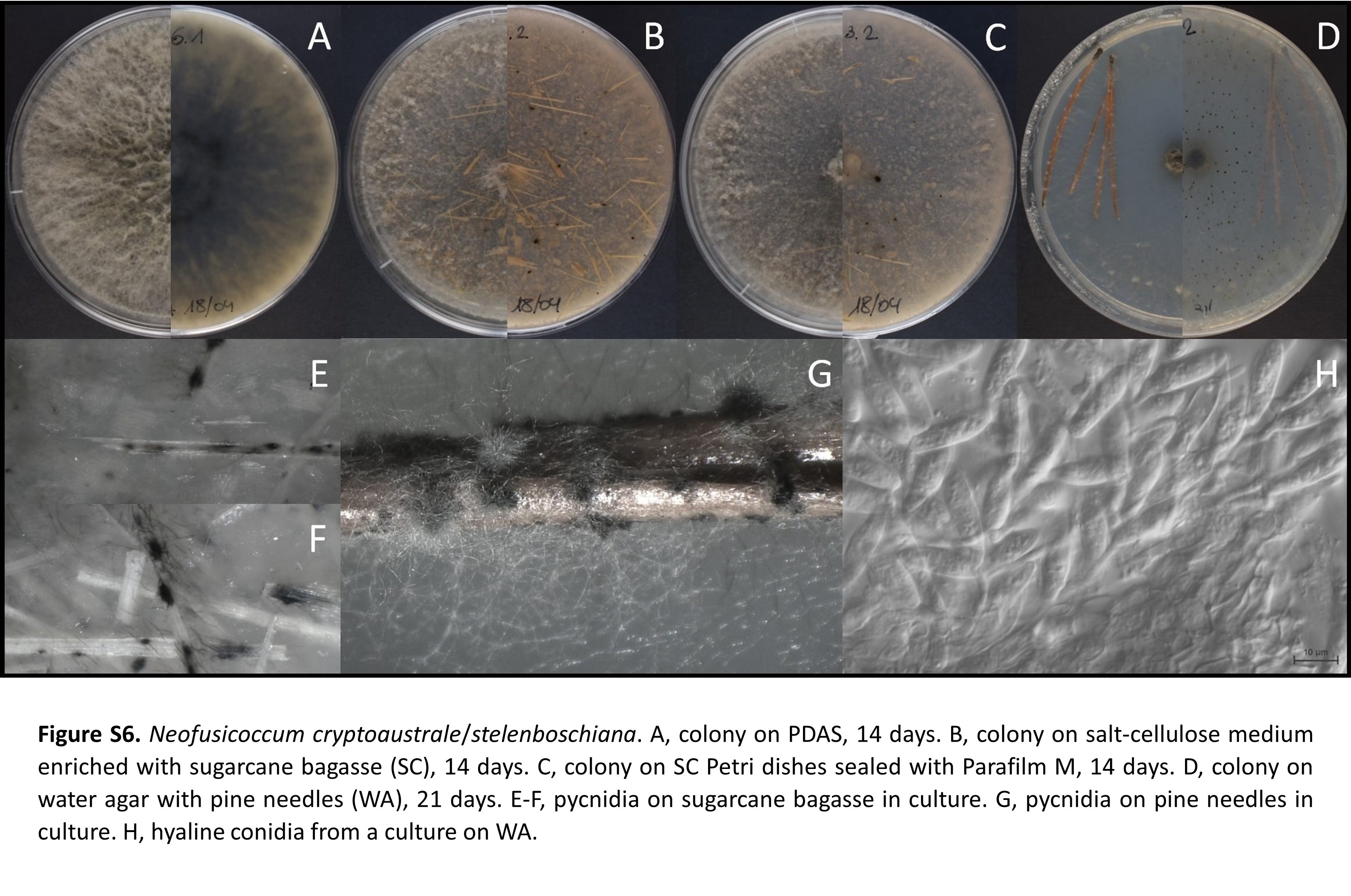

Supplement: Supplementary file 1 [file microorganisms-11-00585-s001.zip › Figure S6.jpg]

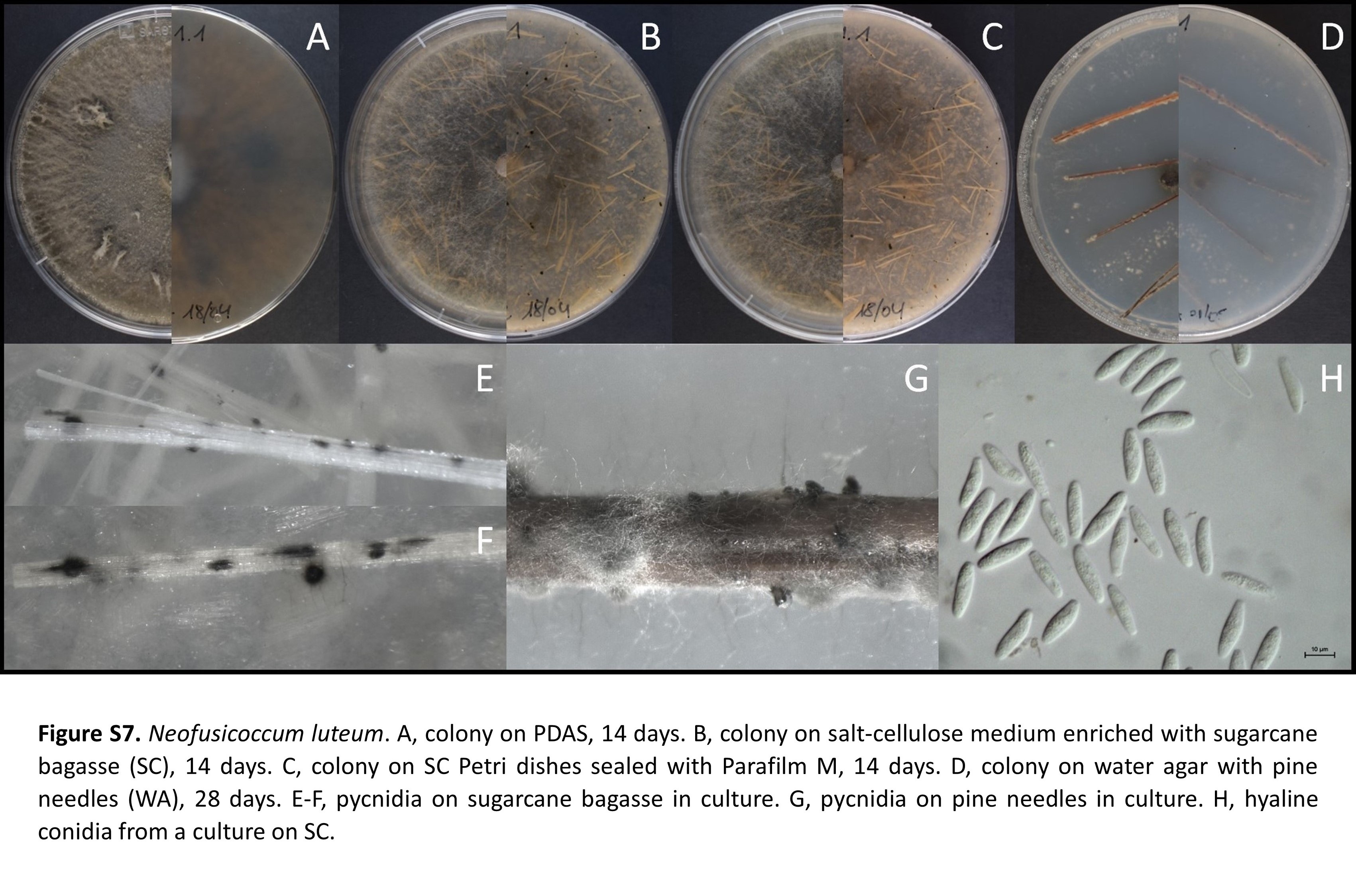

Supplement: Supplementary file 1 [file microorganisms-11-00585-s001.zip › Figure S7.jpg]

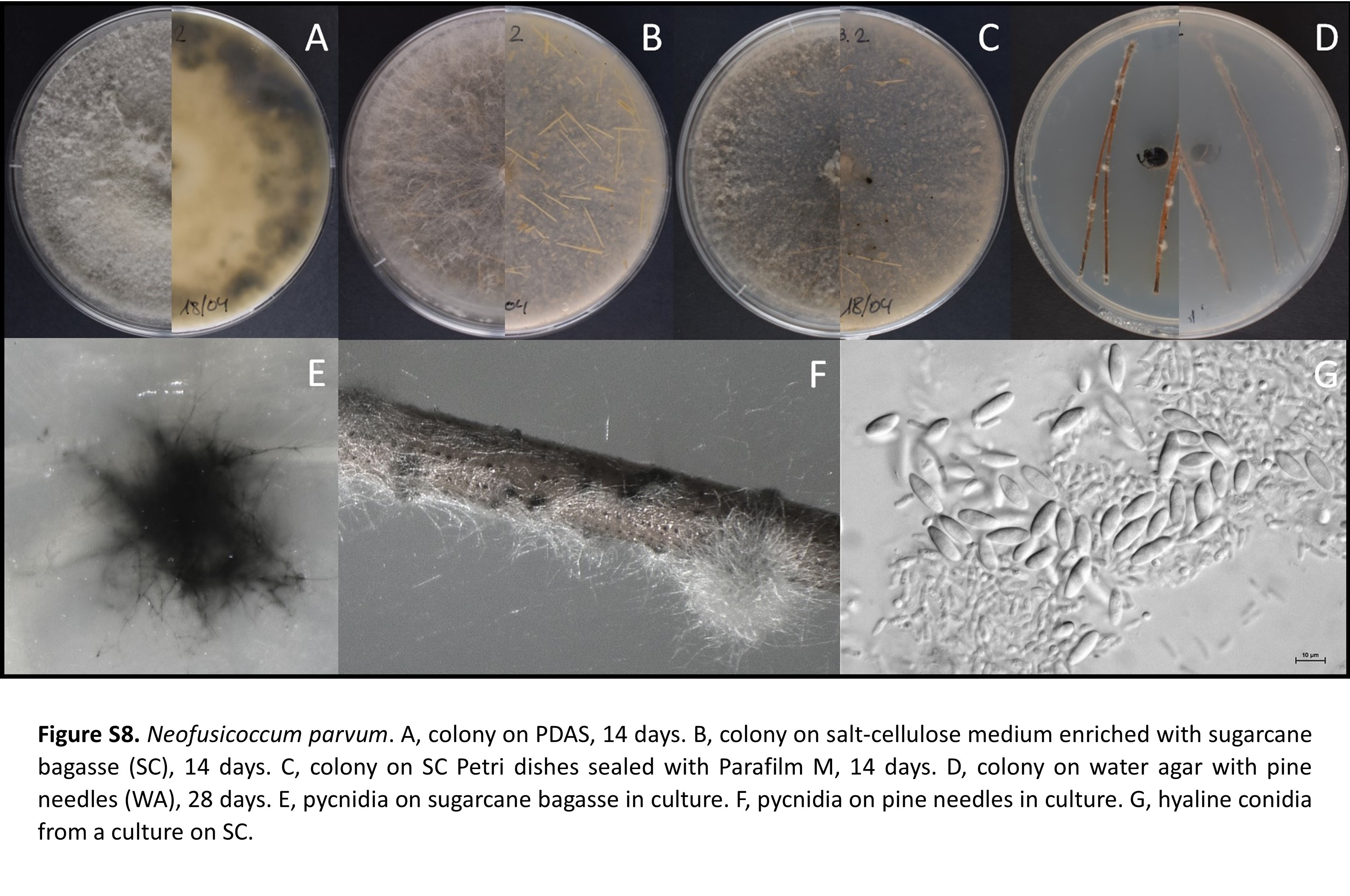

Supplement: Supplementary file 1 [file microorganisms-11-00585-s001.zip › Figure S8.jpg]

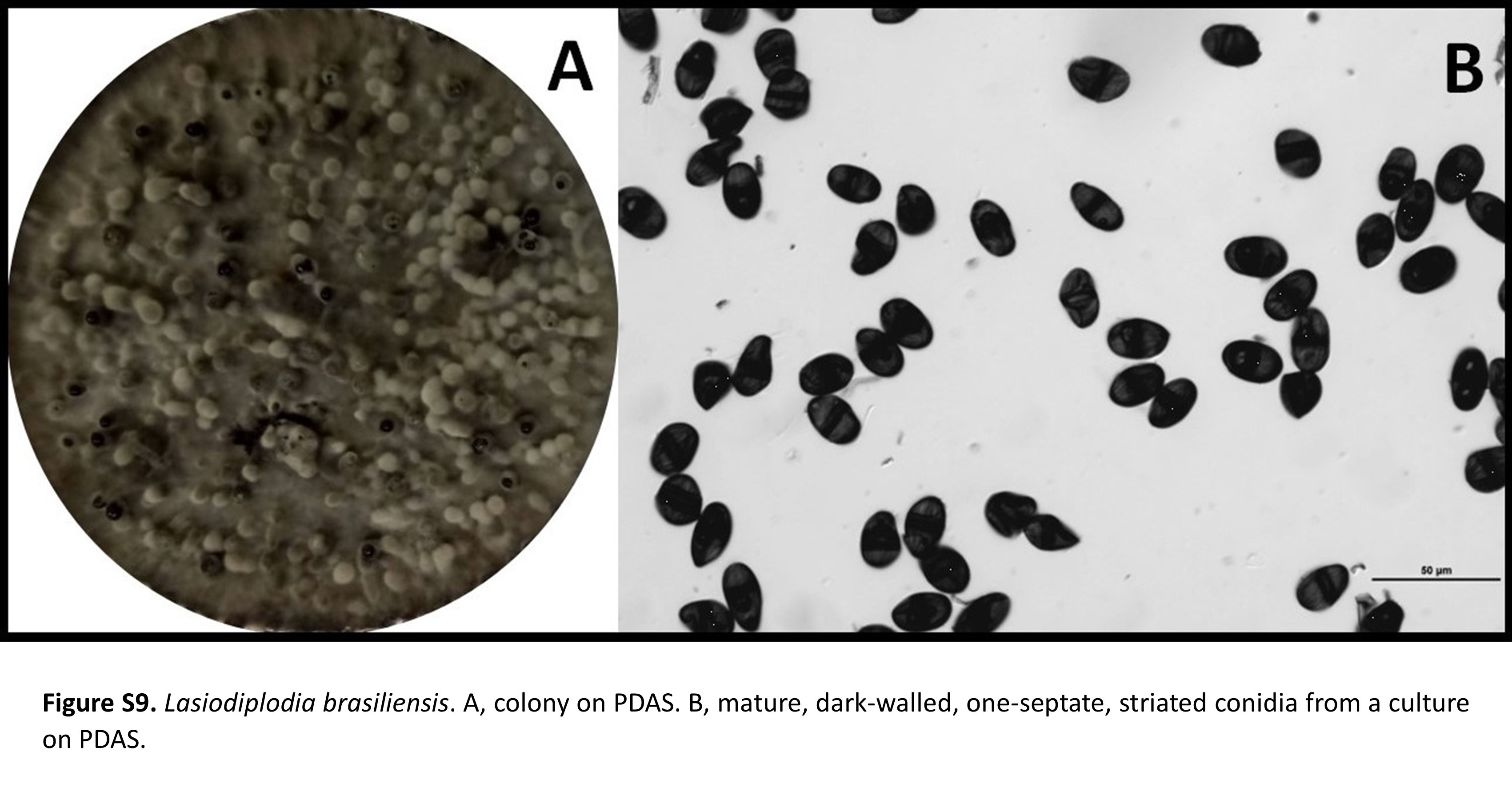

Supplement: Supplementary file 1 [file microorganisms-11-00585-s001.zip › Figure S9.jpg]
